# Predictive and Prognostic Implications of Mutation Profiling and Microsatellite Instability Status in Patients with Metastatic Colorectal Carcinoma

**DOI:** 10.1155/2018/4585802

**Published:** 2018-01-31

**Authors:** Jianhua Liu, Weiqiang Zeng, Chengzhi Huang, Junjiang Wang, Dongyang Yang, Dong Ma

**Affiliations:** ^1^Department of Gastrointestinal Oncology, Cancer Center, Guangdong General Hospital, Guangdong Academy of Medical Sciences, Guangzhou, China; ^2^Department of Pharmacy, Guangdong General Hospital, Guangdong Academy of Medical Sciences, Guangzhou, China; ^3^Department of Gastrointestinal Surgery, Guangdong General Hospital, Guangdong Academy of Medical Sciences, Guangzhou, China

## Abstract

To investigate whether mutation profiling and microsatellite instability (MSI) status were associated with clinicopathological features and the prognosis in metastatic colorectal cancer (mCRC), mutations in *RAS* (including *KRAS*, *NRAS*, and *HRAS*) and *BRAF* were determined by Sanger sequencing. Tumor mismatch repair proteins and MSI status were examined using immunohistochemistry and polymerase chain reaction, respectively. The clinical value of these abnormalities was statistically analyzed, and prognostic value of different treatment regimens was also evaluated. Among 461 mCRC patients, mutations in *RAS*, *BRAF*, and MSI-high (MSI-H) status were observed in 45.3% (209/461), 5.6% (26/461), and 6.5% (30/461) of cases, respectively. Brain metastasis and high carcinoembryonic antigen level were highly correlated with *KRAS* mutation (*P* = 0.011 and *P* < 0.001), and tumors from females or located in the right colon tended to harbor *BRAF* mutation (*P* = 0.039 and *P* = 0.001). *RAS*/*BRAF* mutations may predict brain and/or lung metastases. Although neither clinical nor prognostic importance of MSI status was identified in our study, *KRAS* and *BRAF* mutations were demonstrated to be independent prognostic factors for overall survival and progression-free survival. Besides, in wild-type group, patients treated with chemotherapy plus targeted therapy exhibited the most favorable prognosis. Therefore, *RAS*/*BRAF* mutations may serve as indicators for prognosis and treatment options in mCRC.

## 1. Introduction

Colorectal cancer (CRC) is the third most commonly diagnosed malignancy and the fourth most frequent cause of cancer-associated mortality worldwide [1]. Previous evidence has indicated that liver and lung metastases were quite common in metastatic CRC (mCRC), accounting for approximately 20–30% of all patients when initially diagnosed [2]. As the understanding of molecular mechanisms underlying tumorigenesis and progression of CRC develops, genetic analyses and targeted therapy have already become popular alternatives, representing a significant landmark towards individually tailored treatment.

It is usually admitted that epidermal growth factor receptor (*EGFR*) is an imperative molecular target in mCRC [3]. In general, the monoclonal antibody against *EGFR*, cetuximab or panitumumab, is capable of competitively blocking *EGFR* from binding to its ligand, thus suppressing efficiently downstream RAS/Raf/MAPK pathway activity and improved outcomes [4]. However, mutations of *RAS* (including *KRAS*, *NRAS*, and *HRAS*) and *BRAF* genes may bring about constitutive activation of the pathway, independent of *EGFR* inhibition, which is associated with resistance to anti-*EGFR* therapy [5]. Therefore, the screening of a full gene mutation profiling contributes to select suitable candidates for appropriate therapeutic regimens and regular surveillance.

Microsatellite instability (MSI), a genetic change resulted from mismatch repair (MMR) deficiencies during DNA replication, involves with the pathogenesis of CRC [6]. MSI-high (MSI-H) is known to occur in about 10% of sporadic CRCs and 3% hereditary CRCs [7]. Recently, Le et al. [8] reported a high response rate of mCRC with MSI-H to programmed death-1 (PD-1) inhibitor therapy, indicating that MSI status could be a useful checkpoint for immune therapy.

Multiple researches have documented that *KRAS* mutations were common in a diverse range of human neoplasms, such as lung adenocarcinoma [9], pancreatic cancer [10], and thyroid cancer [11]. Especially in CRC, the rate of *KRAS* mutations is nearly 40%, although *NRAS* or *HRAS* mutations only for less than 3% or 1% [12–14]. Due to high homology and close correlation with *KRAS*, *NRAS* and *HRAS* behave as typical oncogenes [12]. Increasing evidence revealed that CRC patients with *NRAS* mutations had relatively favorable prognosis compared with those with *KRAS* or *BRAF* mutations [15]. However, the clinical importance of *HRAS* mutation remained unclear in CRC because of its rarity [14]. Additionally, as a downstream member of *KRAS*, *BRAF* encodes a serine/threonine protein kinase which plays an important role in cell division and secretion [16]. Cancers with *BRAF* mutation are closely related to tumor location and lower survival, especially for those together with MSI-low (MSI-L) or microsatellite stable (MSS) [17]. Nevertheless, information available about the abnormalities of these oncogenes and the MSI status in mCRC have not been convincingly elucidated.

Here, we comprehensively characterized *RAS*/*BRAF* mutations and MSI status as well as evaluated the prognostic value of different treatment regimens in mCRC patients, which can provide an optimal insight between gene abnormalities and patient survival in Chinese population.

## 2. Materials and Methods

### 2.1. Patients and Clinical Data

The observational model was developed in 461 clinicopathologically confirmed mCRC patients at Guangdong General Hospital (Guangzhou, China) between March 2011 and December 2014. All participants received genetic testing as a part of integrated care. Information on clinicopathological and therapeutic data were obtained from medical archive; tumor classification and grading were based on the World Health Organization criteria. Overall survival (OS) and progression-free survival (PFS) were defined from enrollment start time until death/censoring and tumor progression/censoring, respectively. An outpatient follow-up was conducted every 3 months in accordance with Response Evaluation Criteria in Solid Tumors (RECIST 1.1) in the first 2 years after clinical treatments, followed by every 6 months, until the study endpoint or death. Informed consent was obtained from all individual participants included in the study, and authorization was acquired from the Ethics Committee of Guangdong General Hospital.

### 2.2. Tissue Sampling and Mutation Assessment

Comprehensive genomic profiling was analyzed on 461 formalin-fixed paraffin-embedded (FFPE) primary CRCs retrieved from surgical/endoscopic biopsies and 247 metastases from surgical/percutaneous needle biopsies. Genomic DNA was isolated from each FFPE specimen with QIAamp DNA FFPE Tissue Kit Qiagen (Hilden, Germany) based on the manufacturer's recommendations. Besides that, cancer cell-rich regions were identified in advance by application of hematoxylin-eosin (H&E) staining to ascertain all cases tested enrichment of ≥70% malignant cells. Extracted DNA concentration was determined in a ND-1000 spectrophotometer (Thermo Scientific, Wilmington, DE, USA). Mutations in the *KRAS* (exons 2, 3, and 4), *NRAS* (exons 2, 3, and 4), *HRAS* (exon 2), and *BRAF* (exon 15) of each tumor specimen were examined. AmpliSeq Designer v.1.2.6 software (Life Technologies) was used to design primer pairs for these gene amplifications [18]. DNA amplification was performed by using GoTaq® Hot Start Polymerase (Promega, Madison, WI) and 0.2 lM of each primer with the GeneAmp PCR System 9700 (Applied Biosystems, Foster City, CA) under the cycling conditions as described previously [19]. Amplicons were finally Sanger sequenced bidirectionally on an ABI 3730XL genetic analyzer (Invitrogen Life Technologies, Carlsbad, CA, USA), and detailed procedures were the same as reported earlier [20].

### 2.3. MMR Proteins Determination

Immunohistochemistry (IHC) analysis of the four most frequent MMR proteins (i.e., MLH1, MSH2, MSH6, and PMS2) was conducted on FFPE tumor specimens following standard IHC protocols [21]. Representative tumor areas were carefully selected and marked before paraffin blocks were longitudinally sliced to 4 *μ*m thick sections. Immunostaining was carried out with mouse monoclonal antibodies MLH1 (liquid, 1 : 150 dilution; BD, New Jersey, USA), MSH2 (lyophilized, 1 : 100 dilution; BD, New Jersey, USA), MSH6 (liquid, 1 : 150 dilution; BD, New Jersey, USA), and PMS2 (liquid, 1 : 150 dilution; BD, New Jersey, USA). Normal protein expression presented nuclear staining of tumor cells, while negative result showed no nuclear staining in tumor cells with concurrent positive controls within surrounding cells. Tumors were classified as MMR deficiency (MMR-D) when any MMR protein expression was negative and MMR intact (MMR-I) when all MMR proteins were positively expressed. The results were judged by two independent pathologists.

### 2.4. Analysis of Microsatellite Instability (MSI) Status

Extracted DNA samples from primary CRCs and paired metastases were also used for MSI analysis. Briefly, MSI status was examined with the panel of five microsatellite markers (BAT25, BAT26, NR21, NR24, and NR27) by fluorescence-based PCR. Primer pairs for amplification were designed using the software package mentioned above. DNA was amplified in a 20 *μ*L volume with GoTaq Hot Start Polymerase (Promega, Madison, WI), starting with an initial 5-minute denaturation step at 95°C, then 35 cycles at 95°C for 30 seconds, annealing at 60°C for 30 seconds, and extension at 72°C for 30 seconds and finally an extension at 72°C for 10 minutes. The PCR products were analyzed on a Genetic Analyzer (Applied Biosystems 3500, ABI), and allelic sizes were determined with the GeneMapper Software (Applied Biosystems). Patients were defined as MSI-L if a single marker presented instability, MSI-H if two or more of the five studied markers showed instability, and MSS if no marker showed instability.

### 2.5. Statistical Analysis

The data analysis was performed by SPSS version 19.0 (SPSS Inc., Chicago, IL, USA). The correlation between gene status and clinicopathological variables was compared with Pearson's Chi-square (*χ* ^2^) test. Logistic regression was done to identify potential predictors for brain/lung metastases, and the area under the receiver operating characteristic (ROC) was used to estimate the predictive value of the clinical factors. Survival curves were plotted by Kaplan-Meier method with a log-rank test. Univariate and multivariate proportional Cox models were employed to assess independent prognostic factors. The statistically significant difference was set at 0.05.

## 3. Results

### 3.1. Frequency of Gene Mutations in Primary Lesions and Corresponding Metastases

Among 461 primary CRCs, 231 (50.1%) were *RAS*/*BRAF* wild-type. *KRAS*, *NRAS*, and *HRAS* mutations were observed in 43.6% (201/461), 2.8% (13/461), and 0.2% (1/461) of cases, respectively. Besides, as another indispensible incidence of *EGFR* pathway, *BRAF* mutations were present in 5.6% (26/461) cases. Notably, gene mutations in primary lesions were highly coincident with those in matched metastases except two patients, whose *KRAS* mutations occurred in primary tumors rather than metastases. The most frequently noted mutation occurred in exon 2 (codons 12 and 13) of *KRAS* (37.1%, 171/461). Detailed distribution of mutation subtypes was summed up in Table 1.

**Figure 1:**
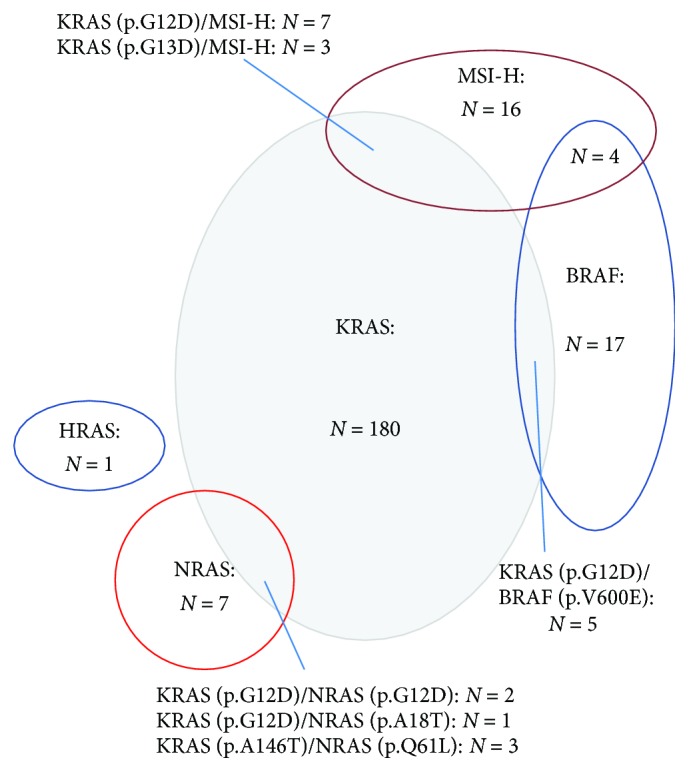
Set diagram illustrates the associations among *KRAS*, *NRAS*, HRAS, and *BRAF* mutations and MSI-H status. Mutations in *KRAS* and *NRAS* are not mutually exclusive, and neither are *KRAS* and *BRAF*. MSI-H status cooccurred with *KRAS* or *BRAF* mutations. MSI-H: microsatellite instability-high.

Particularly, mapping correlations between different gene mutations demonstrated that 6 patients carried both *KRAS* and *NRAS* mutations, and in another 5 patients, *KRAS* and *BRAF* mutations concomitantly existed. However, no cooccurring mutations of *NRAS* with *BRAF* were observed in our study, nor did *HRAS* and other genes (Figure 1).

### 3.2. Frequency of Loss of MMR Protein Expression and MSI Status Detection

**Figure 2:**
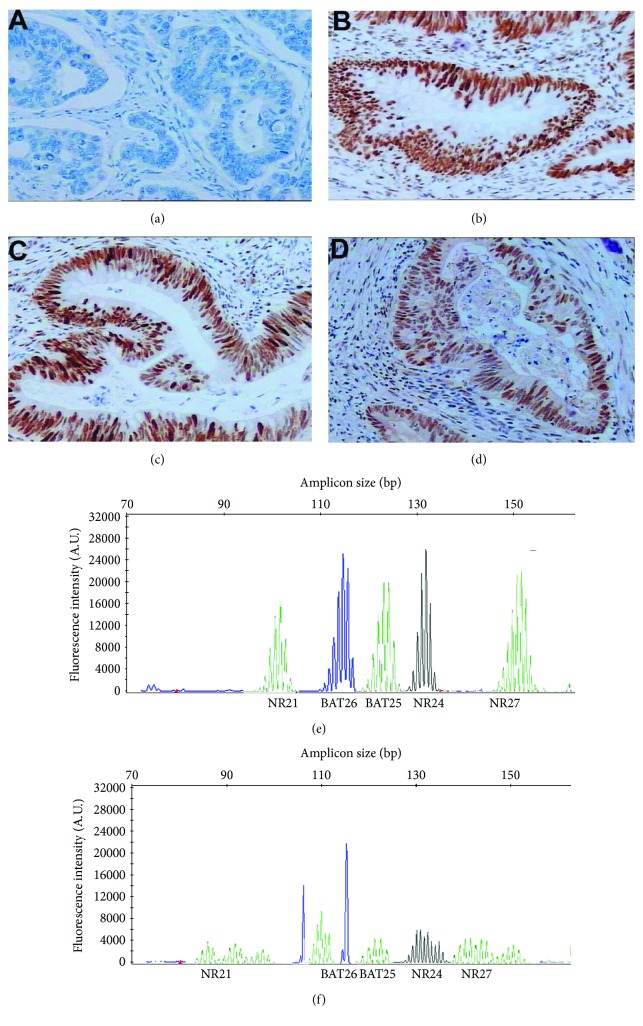
MMR protein determination and MSI status analysis. Immunohistochemical staining pattern of MSI-L colorectal carcinoma samples with isolated loss of MLH1 (a) and intact staining of MSH2 (b), MSH6 (c), and PMS2 (d); examples of fluorescence-based PCR of mononucleotide repeats and typical profiles of a MSS tumor (e) and a MSI-H case (f). MMR: mismatch repair; MSI: microsatellite instability; MSI-L: MSI-low; MSI-H: MSI-high; MSS: microsatellite stability; PCR: polymerase chain reaction.

Among the entire study population, 32 cases (6.9%) were MMR-D phenotype, while 429 cases (93.1%) were MMR-I phenotype in primary CRCs. In the MMR-D cases, MLH1 expression loss was the most common (46.9%, 15/32) (Figures 2(a)–2(d)). Moreover, the specimens were also tested by PCR, the gold standard for confirming MSI status. Results showed that 30 primary tumors (6.5%) were with MSI-H, 45 (9.8%) were with MSI-L, and 386 (83.7%) were with MSS (Figures 2(e) and 2(f)). Similarly, there was a high concordance of MMR protein expression (98.8%, 244/247) and MSI status (98.4%, 243/247) between primary lesions and corresponding metastases. Specifically, three cases carrying MMR-I primary lesions exhibited the MMR-D phenotype in metastases. Of the four discordant cases with MSS primary tumors, three carried MSI-L metastases and one carried MSI-H metastases. Besides, MSI-H and *KRAS*/*BRAF* mutations can coexist according to our data (Figure 1).

### 3.3. Clinical Significance of RAS/BRAF Mutations and the MSI Status in mCRC Patients

All analyses were carried out in terms of sequencing outcomes in primary lesions. *KRAS* mutations were closely correlated with brain metastasis (*P* = 0.011) and high carcinoembryonic antigen (CEA) level (*P* < 0.001), and *BRAF* revealed a higher mutation rate in female patients (*P* = 0.039) and the right colon (*P* = 0.001). As for *NRAS* mutations, no significant relevance with the characteristics was observed. *HRAS* mutation was too rare to further explore. Moreover, no remarkable association between MSI status and gene mutations was identified in our study (*P* > 0.05) (Table 2).

### 3.4. Predictors of Brain and/or Lung Metastases according to the Clinical Factors

Unconditional logistic regression revealed that *RAS*/*BRAF* mutations and moderate/strong C-MET expression were both significantly correlated with the occurrence of brain and/or lung metastases [odds ratio (OR): 4.027, *P* < 0.001 and OR: 3.901, *P* < 0.001, respectively (Table 3)].

**Figure 3:**
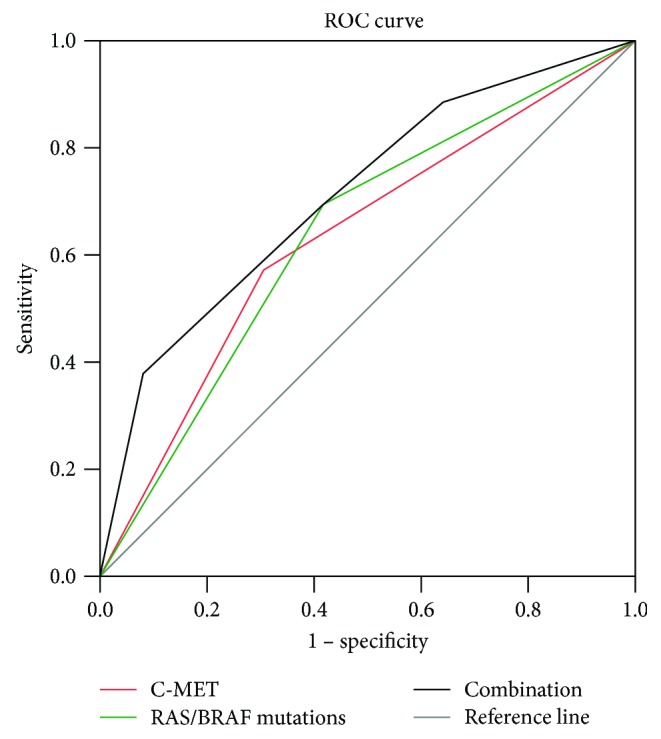
ROC curves for the predictive value of *RAS*/*BRAF* mutations and C-MET expression for brain and/or lung metastasis. ROC: receiver operating characteristic curve.

With ROC curve analysis, the sensitivity and specificity of *RAS*/*BRAF* mutations alone, C-MET expression alone, or their combination for predicting brain and/or lung metastases within mCRC patients were evaluated. The findings which indicated a combination of *RAS*/*BRAF* mutations and C-MET expression [area under curve (AUC): 0.711, 95% CI: 0.659–0.763, *P* < 0.001] exhibited a better predictive value compared with single *RAS*/*BRAF* mutations (AUC: 0.638, 95% CI: 0.584–0.693, *P* < 0.001) or C-MET expression (AUC: 0.634, 95% CI: 0.578–0.690, *P* < 0.001) (Figure 3).

### 3.5. Survival Analysis

**Figure 4:**
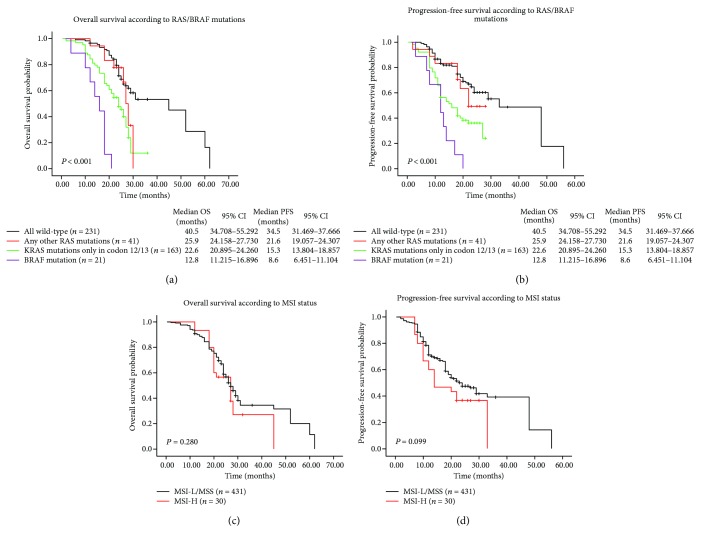
Kaplan-Meier survival curves of metastatic colorectal carcinoma patients. (a) OS and (b) PFS of patients with different gene mutations; (c) OS and (d) PFS (MSI-L/MSS versus MSI-H) of entire study population. OS: overall survival; PFS: progression-free survival; MSI: microsatellite instability; MSI-L: MSI-low; MSI-H: MSI-high; MSS: microsatellite stability.

By the cutoff day on October 1, 2017, 257 (56.2%) of the enrolled patients had demised during the follow-up period. The median follow-up period was 24.3 months (range, 0.6–62 months), while 24 (5.2%) patients lost to follow-up. The potential influence of gene mutations and MSI status on survival was assessed with the Kaplan-Meier method. It was concluded that OS and PFS for patients with *RAS*/*BRAF* mutations were significantly shortened than those of cases with all wild-type. Particularly, cases exhibiting *BRAF* mutations had the worst prognosis (median OS and PFS: 12.8 months and 8.6 months), instead the any-other-*RAS*-mutated group had longer median OS and PFS (25.9 months and 21.6 months) than the other two mutational groups (Figures 4(a) and 4(b)). However, patients with different MSI status did not significantly differ in OS and PFS (*χ* ^2^ = 1.165, *P* = 0.280 and *χ* ^2^ = 2.717, *P* = 0.099; Figures 4(c) and 4(d)).

Furthermore, clinical value of various prognostic factors was estimated using Cox proportional hazards model. As confirmed by multivariate analyses, *KRAS* or *BRAF* mutation emerged as an independent risk factor for OS [hazard ratio (HR): 1.826, 95% confidence interval (CI): 1.361–2.450, *P* < 0.001 and HR: 4.798, CI: 2.989–7.700, *P* < 0.001; Table 4] and PFS (HR: 2.082, CI: 1.545–2.805, *P* < 0.001 and HR: 3.864, CI: 2.375–6.287, *P* < 0.001). In brief, our findings revealed that *RAS*/*BRAF* mutations played an essential role in patients' survival.

### 3.6. Prognostic Value of Different Treatment Regimens and Efficacy of Anti-EGFR Therapies

**Figure 5:**
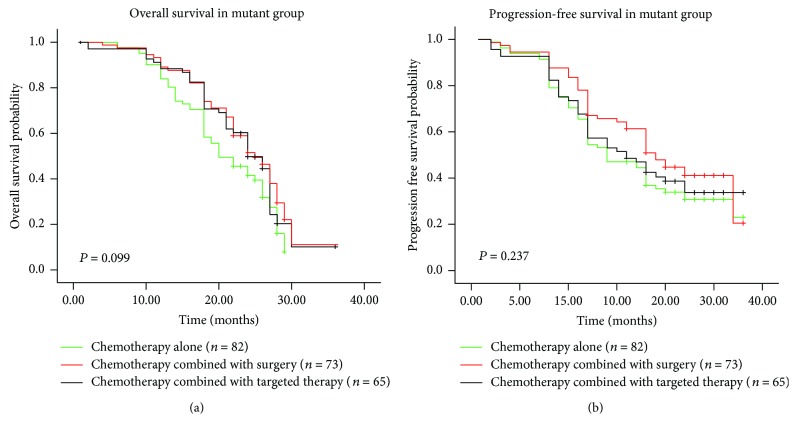
Kaplan-Meier survival curves of mutant group stratified according to treatment regimens. (a) OS and (b) PFS of patients treated with different regimens. OS: overall survival; PFS: progression-free survival.

**Figure 6:**
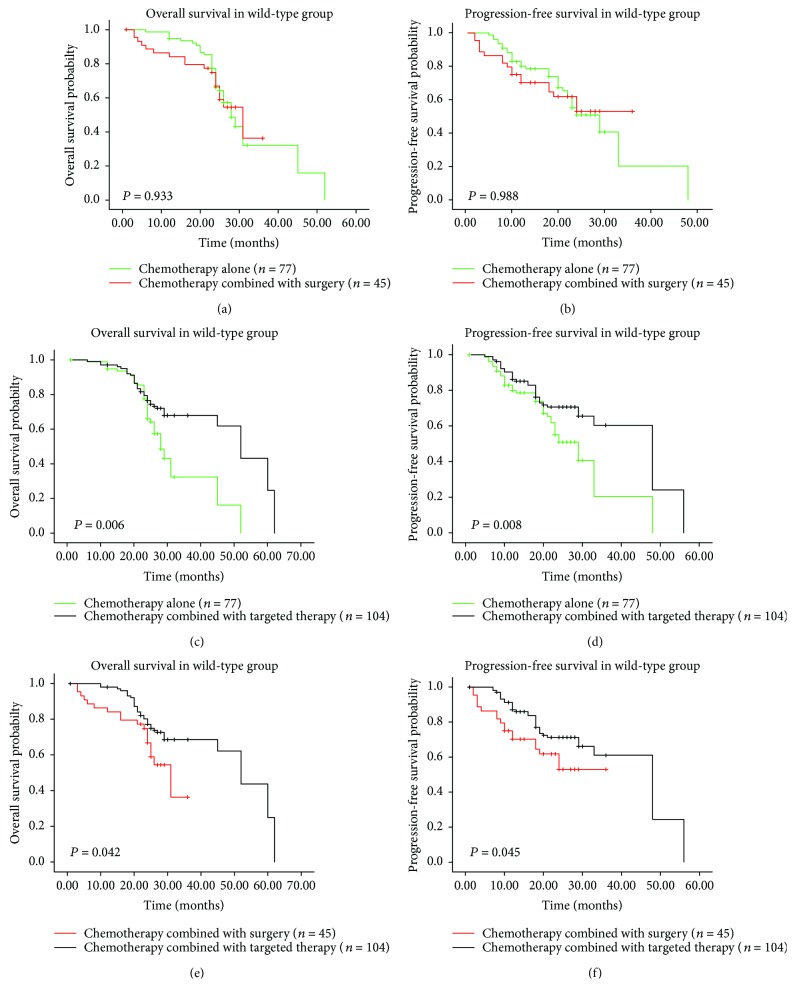
Kaplan-Meier survival curves of wild-type group stratified according to treatment regimens. (a) OS and (b) PFS chemotherapy alone versus chemotherapy combined with surgery, (c) OS and (d) PFS chemotherapy alone versus chemotherapy combined with targeted therapy, and (e) OS and (f) PFS chemotherapy combined with surgery versus chemotherapy combined with targeted therapy. OS: overall survival; PFS: progression-free survival.

Of 461 mCRC patients, 452 (98.0%) received oxaliplatin-based or irinotecan-based chemotherapy, including 159 cases treated with chemotherapy alone, 118 combined with surgery, and 169 combined with targeted therapies (Table 5). Further analyses revealed in *RAS*/*BRAF* mutant group, different treatment regimens showed no significant difference on OS and PFS (*χ* ^2^ = 4.621, *P* = 0.099 and *χ* ^2^ = 2.882, *P* = 0.237; Figures 5(a) and 5(b)). In contrast, among wild-type patients, chemotherapy plus targeted therapies exhibited more favorable prognosis than the other treatment options (Figures 6(c)–6(f)), although there was no significant difference on survival (OS: *χ* ^2^ = 0.007, *P* = 0.933; PFS: *χ* ^2^ = 0.001, *P* = 0.988; Figures 6(a) and 6(b)) between chemotherapy alone and chemotherapy plus surgery groups. Moreover, bevacizumab therapy has been confirmed to be an independent prognostic factor for improved outcomes (Table 4).

Among wild-type participants, 48 were treated by chemotherapy plus anti-*EGFR* agents. Data showed that the disease control rate (DCR) was 72.9% (35/48), with no patient for complete response (CR), 11 patients for partial response (PR), and 24 cases for stable disease (SD) for the first response evaluation at 3 months. In addition, 4 subjects with gene mutations also received cetuximab treatment (1 with *BRAF* mutation and 3 with *KRAS* exon 4 mutation), but DCR was 0.0% (0/4). Thus, the DCR and the response rate (including CR and PR) of wild-type patients were relatively better than those of cases with *RAS*/*BRAF* mutations (72.9% versus 0.0% and 22.9% versus 0.0%), although no statistical significance was attained.

## 4. Discussion

As a pathologically and clinically heterogeneous malignancy, CRC presented high aggressiveness and an accompanying worse prognosis on account of its aggressive nature. Despite the complexity of carcinogenesis, the discovery of extensive molecular markers for CRC has attracted special interests. As a result, gene detection has been attached to important connections with CRC evaluation and targeted therapy. However, the predictive and prognostic value of *RAS*/*BRAF* mutations and the MSI status in human mCRC has not previously been comprehensively elucidated.

Based on our data, the prevalence of gene mutations or the MSI-H status was in line with previous publications [12, 13, 22–24]. Meanwhile, there was a high concordance between primary CRCs and corresponding metastases, demonstrating that *RAS*/*BRAF* abnormalities emerged early in CRC tumorigenesis [25], and tumor cells kept their MSI status during development [6]. Different from intratumoral heterogeneity of *KRAS* mutations and rare *NRAS* or *HRAS* mutation, *BRAF* mutation showed relative intratumoral homogeneity [26, 27]. In addition, the present study also demonstrated that mutations in *RAS*/*BRAF* were not mutually exclusive, although the finding conflicted with several studies from other populations [28, 29]. One likely explanation may be the disparity of included cases and sample sources (Chinese versus European population). Regarding the MSI status, Fujiyoshi et al. [6] proposed that MSI-H status and *RAS*/*BRAF* mutations could coexisted. Similarly, our results corroborated the fact. Given increasing data on mutation profiling was accumulated, associations among *RAS*/*BRAF* genes will be further expounded.

Moreover, we characterized *RAS*/*BRAF* mutations and MSI status, and results revealed that *RAS* or *BRAF* mutation possessed clinical significance in promoting the development and metastasis of mCRC. In brief, *KRAS* mutations may be important indicators to identify subsets with increased CEA level and brain metastases. The viewpoints were partially different from literatures published, in which *KRAS* mutations were related to older age, differentiation degree, and later clinical stage [22, 29, 30]. The variability in various researches probably attributed to geographical distribution and ethnicities. Until now, the significance of *NRAS* or *HRAS* mutations remained controversial due to their rarity. A recent CRC study [12] proposed that *NRAS* mutations were found to be tilted to the right colon and MSI-L cancers. Nevertheless, no clinical relevance of *NRAS* mutations was observed in our research; *HRAS* mutation was too rare to further explore. Recently, Zhang et al. [26] reported that *BRAF* mutations were observed more frequently in the right colon and female patients, which supported the conclusions of our study. Particularly, no significant association was found between the MSI status and *RAS*/*BRAF* mutations, albeit a recent report [6] showed that MSI-H linked with *BRAF* mutations. This bias might be caused by the limited data and the different detection techniques.

The initiation and progression of CRC are a multistep process accompanied by inactivation of tumor suppressors and accumulation of gene mutations, especially somatic changes in *RAS*/*BRAF*, which are driver mutations and represent the principle aspect of gene abnormalities in CRC [31]. Another focus of our research was searching for the predictive value of *RAS*/*BRAF* mutations and MSI status. Numerous experimental model systems have confirmed that *RAS*/*BRAF* abnormalities contributed to cell invasion and apoptosis suppression during metastatic cascade, which may bring about organ involvement and tumor progression [4, 32]. In one previous study [33], *KRAS* exon 2-mutated CRC patients exhibited an obvious propensity for lung metastases. Similar results have also been described by Morris et al. [34], in which cases with *RAS*/*BRAF* mutations harbored the trend towards lung metastases. Here, our data for the first time revealed that *RAS*/*BRAF* mutations were significant predictors for higher risk of brain metastases, followed by lung metastases, suggesting its value in distinguishing CRC with highly aggressive behavior from low metastatic ability. Thus, the emergence of *RAS*/*BRAF* mutations provided powerful insight into the complexity of tumor foci genotype and gained useful clues for treatment option.

Unfortunately, when it came to the MSI status, neither predictive nor prognostic relevance was observed in mCRC. This phenomenon was concordant with studies issued [24]. But for stage II or III cases, MSI-H contributed to the favorable prognosis [7]. Because of too few MSI-H cases restrained the discovery of potential clinical and prognostic value of MSI status, more focusing on the issue was desired.

Mutation in *KRAS* was regarded as an adverse predictors for disease-specific survival more early in 1990 [35]. Not until the last ten years, prognostic ability of *RAS*/*BRAF* aberrations in CRC has spurred much more attention. In agreement with previous series [15, 34], our data also revealed that patients with gene mutations, especially *BRAF* mutation, suffered inferior prognosis compared with wild-type counterparts. Interestingly, cases carrying *NRAS* mutations showed relatively better survival than those with other *RAS* mutations. Besides that, as the National Comprehensive Cancer Network (NCCN) recommends targeted therapies for mCRC patients, our analysis suggested that chemotherapy combined with targeted therapy could remarkably improve the prognosis of wild-type patients. Importantly, bevacizumab had been considered as an independent prognostic factor according to our data, which accorded with some meta-analyses and randomized controlled trials [36, 37]. Meantime, *RAS*/*BRAF* mutations were emphasized to be predictive biomarkers of resistance to therapies against *EGFR*, and only wild-type CRC patients may gain survival benefit from cetuximab and panitumumab.

Owing to the retrospective nature, there have been inevitably selection bias in our outcomes. Firstly, some participants and their medical record documentation may be lost to follow-up, especially for those who were not hospitalized after first-line chemotherapy. Secondly, the patients were heterogeneous and selected according to availability of molecular detection, which limited the data analyses. Therefore, more prospective studies are required to confirm our conclusions.

## 5. Conclusions

Altogether, *RAS*/*BRAF* mutations may serve as significant predictors of malignant behavior. Accordingly, radiological diagnosis combined with gene detection may help to evaluate the prognosis of novel CRC cases and devised optimal individualized medicine in the future.

## Figures and Tables

**Table 1:** Mutation subtype frequency distribution of RAS and BRAF genes.

Genes	Codon	Mutation	Cases (% of 461)
Total cases with RAS mutation			230 (49.9%)
Total cases with KRAS mutation			201 (43.6%)
KRAS	12	p.G12D	74 (16.1%)
	12	p.G12V	35 (7.6%)
	12	p.G12C	15 (3.3%)
	12	p.G12A	6 (1.3%)
	12	p.G12R	9 (1.9%)
	12	p.G12S	1 (0.2%)
	13	p.G13D	31 (6.7%)
	59	p.A59T	4 (0.9%)
	61	p.Q61H	2 (0.4%)
	146	p.A146T	16 (3.5%)
	146	p.A146V	3 (0.6%)
	Others	Others	5 (1.1%)
Total cases with NRAS mutation			13 (2.8%)
NRAS	12	p.G12D	4 (0.9%)
	12	p.G12S	2 (0.4%)
	18	p.A18T	1 (0.2%)
	61	p.Q61L	4 (0.9%)
	61	p.Q61R	2 (0.4%)
Total cases with HRAS mutation			1 (0.2%)
HRAS	12	p.G12D	1 (0.2%)
Total cases with BRAF mutation			26 (5.6%)
BRAF	600	V600E	26 (5.6%)

**Table 2:** Correlation between mutation profile and clinicopathological features in 461 patients with metastatic colorectal cancer.

Clinicopathological features	*n*	KRAS	Status		BRAF	Status		NRAS	Status		All wild-type	Any mutation	
		Wild-type (*n* = 260, %)	Mutation (*n* = 201, %)	*P*	Wild-type (*n* = 435, %)	Mutation (*n* = 26, %)	*P*	Wild-type (*n* = 448, %)	Mutation (*n* = 13, %)	*P*	(*n* = 231, %)	(*n* = 230, %)	*P*
Gender													
Male	250	143 (57.2)	107 (42.8)	0.706	241 (96.4)	9 (3.6)	**0.039**	245 (98.0)	5 (2.0)	0.247	133 (53.2)	117 (46.8)	0.148
Female	211	117 (55.5)	94 (44.5)		194 (91.9)	17 (8.1)		203 (96.2)	8 (3.8)		98 (46.4)	113 (53.6)	
Age, years													
<65	241	140 (58.1)	101 (41.9)	0.443	231 (95.9)	10 (4.1)	0.147	237 (98.3)	4 (1.7)	0.115	131 (54.4)	110 (45.6)	0.056
≥65	220	120 (54.5)	100 (45.5)		204 (92.7)	16 (7.3)		211 (95.9)	9 (4.1)		100 (45.5)	120 (54.5)	
Tumor location													
Left colon	209	123 (58.9)	86 (41.1)	0.509	193 (92.3)	16 (7.7)	**0.001**	205 (98.1)	4 (1.9)	0.097	105 (50.2)	104 (49.8)	0.565
Right colon	55	32 (58.2)	23 (41.8)		48 (87.3)	7 (12.7)		51 (92.7)	4 (7.3)		24 (43.6)	31 (56.4)	
Rectum	197	105 (53.3)	92 (46.7)		194 (98.5)	3 (1.5)		192 (97.5)	5 (2.5)		102 (51.8)	95 (48.2)	
Primary tumor size													
<5 cm	381	216 (56.7)	165 (43.3)	0.781	357 (93.7)	24 (6.3)	0.181	368 (96.6)	13 (3.4)	0.094	189 (49.6)	192 (50.4)	0.638
≥5 cm	80	44 (55.0)	36 (45.0)		78 (97.5)	2 (2.5)		80 (100.0)	0 (0.0)		42 (52.5)	38 (47.5)	
Differentiation													
Well/moderate	289	166 (57.4)	123 (42.6)	0.559	269 (93.1)	20 (6.9)	0.122	278 (96.2)	11 (3.8)	0.097	142 (49.1)	147 (50.9)	0.588
Poor	172	94 (54.7)	78 (45.3)		166 (96.5)	6 (3.5)		170 (98.8)	2 (1.2)		89 (51.7)	83 (48.3)	
Histological type													
Papillary/tubular adenocarcinoma	380	211 (55.5)	169 (44.5)	0.413	358 (94.2)	22 (5.8)	0.763	369 (97.1)	11 (2.9)	0.834	186 (48.9)	194 (51.1)	0.280
Mucinous/signet ring cell	81	49 (60.5)	32 (39.5)		77 (95.1)	4 (4.9)		79 (97.5)	2 (2.5)		45 (55.6)	36 (44.4)	
Depth of invasion													
T1	2	0 (0.0)	2 (100.0)	0.266	2 (100.0)	0 (0.0)	0.557	2 (100.0)	0 (0.0)	0.53	0 (0.0)	2 (100.0)	0.300
T2	30	14 (46.7)	10 (53.3)		28 (93.3)	2 (6.7)		30 (100.0)	0 (0.0)		12 (40.0)	18 (60.0)	
T3	347	158 (57.6)	126 (42.4)		325 (93.7)	22 (6.3)		335 (96.5)	12 (3.5)		175 (50.4)	172 (49.6)	
T4	82	38 (56.1)	26 (43.9)		80 (97.6)	2 (2.3)		81 (98.8)	1 (1.2)		44 (53.7)	38 (46.3)	
Nodal stage													
N0	32	15 (46.9)	17 (53.1)	0.143	32 (100.0)	0 (0.0)	0.319	32 (100.0)	0 (0.0)	0.680	15 (46.9)	17 (53.1)	0.224
N1	285	171 (60.0)	114 (40.0)		265 (93.0)	20 (7.0)		276 (96.8)	9 (3.2)		150 (46.9)	135 (53.1)	
N2a	95	52 (54.7)	43 (45.3)		91 (95.8)	4 (4.2)		93 (97.9)	2 (2.1)		48 (50.5)	47 (49.5)	
N2b	49	22 (44.9)	27 (55.1)		47 (95.9)	2 (4.1)		47 (95.9)	2 (4.1)		18 (36.7)	31 (63.3)	
Metastatic site													
Brain	19	4 (21.1)	15 (78.9)	**0.011**	17 (89.5)	2 (10.5)	0.172	19 (100.0)	0 (0.0)	0317	3 (15.8)	16 (84.2)	**0.002**
Lung	121	66 (54.5)	55 (45.5)		112 (92.6)	9 (7.4)		115 (95.0)	6 (5.0)		53 (43.8)	68 (56.2)	
Liver	233	136 (58.4)	97 (41.6)		219 (94.0)	14 (6.0)		227 (97.4)	6 (2.6)		122 (52.4)	111 (47.6)	
Others	88	54 (61.4)	34 (38.6)		87 (98.9)	1 (1.1)		87 (98.9)	1 (1.1)		53 (60.2)	35 (39.8)	
COX-2 expression													
Negative	128	63 (49.2)	65 (50.8)	0.054	123 (96.1)	5 (3.9)	0.317	124 (96.9)	4 (3.1)	0.806	58 (45.3)	70 (54.7)	0.202
Positive	333	197 (59.2)	136 (40.8)		312 (93.7)	21 (6.3)		324 (97.3)	9 (2.7)		173 (52.0)	160 (48.0)	
C-MET expression													
Negative/weak	178	109 (61.2)	69 (38.8)	0.097	171 (96.1)	7 (3.9)	0.208	171 (96.1)	7 (3.9)	0.252	99 (55.6)	79 (44.4)	0.061
Moderate/strong	283	151 (53.4)	132 (46.6)		264 (93.3)	19 (6.7)		277 (97.9)	6 (2.1)		132 (46.6)	151 (53.4)	
Initial CEA (ng/mL)													
<20	70	54 (77.1)	16 (22.9)	<**0.001**	68 (97.1)	2 (2.9)	0.273	70 (100.0)	0 (0.0)	0.122	52 (74.3)	18 (25.7)	<**0.001**
≥20	391	206 (52.7)	185 (47.3)		367 (93.9)	24 (6.1)		378 (96.7)	13 (3.3)		179 (45.8)	212 (54.2)	
MSI													
MSI-H	30	20 (66.7)	10 (33.3)	0.241	26 (86.7)	4 (13.3)	0.059	30 (100.0)	0 (0.0)	0.335	16 (53.3)	14 (46.7)	0.715
MSI-L/MSS	431	240 (55.7)	191 (44.3)		409 (94.9)	22 (5.1)		418 (97.0)	13 (3.0)		215 (49.9)	216 (50.1)	

COX-2 = cyclooxygenase-2; CEA = carcinoembryonic antigen; C-MET = mesenchymal-epithelial transition factor; MSI = microsatellite instability; MSI-H = MSI-high; MSI-L = MSI-low; MSS = microsatellite stability.

**Table 3:** Logistic regression analysis of the factors associated with brain and/or lung metastases in metastatic colorectal cancer patients.

Characteristics	OR	95% CI	*P* value
C-MET expression: negative/weak versus moderate/strong	3.901	2.496–6.098	<0.001
RAS/BRAF genes: all wild-type versus any mutation	4.027	2.551–6.358	<0.001
Constant	0.111		

*P* < 0.05 is statistically significant. CI: confidence interval; OR: odds ratio; C-MET: mesenchymal-epithelial transition factor.

**Table 4:** Univariate and multivariate analyses of OS and PFS for 461 metastatic colorectal cancer patients.

Parameter	Variable	OS univariate	Analysis	OS multivariate	Analysis	PFS univariate	Analysis	PFS multivariate	Analysis
		HR (95% CI)	*P* value	HR (95% CI)	*P* value	HR (95% CI)	*P* value	HR (95% CI)	*P* value
Gender	Male versus female	0.998 (0.773–1.287)	0.985			1.026 (0.795–1.325)	0.841		
Age, years	<65 versus ≥65	1.310 (1.013–1.694)	**0.039**	1.330 (0.998–1.772)	0.052	1.067 (0.827–1.376)	0.619		
Tumor location	Left/right colon versus rectum	1.013 (0.884–1.162)	0.849			1.089 (0.950–1.249)	0.220		
Primary tumor size, cm	<5 versus ≥5	1.077 (0.783–1.480)	0.650			1.311 (0.955–1.800)	0.094		
Differentiation	Well/moderate versus poor	1.031 (0.790–1.345)	0.823			1.322 (1.016–1.721)	**0.038**	0.395 (0.145–1.075)	0.069
Histological type	Papillary/tubular adenocarcinoma versus mucinous/signet ring cell	1.142 (0.829–1.575)	0.416			1.280 (0.929–1.764)	0.131		
Depth of invasion	T1 + T2 versus T3 + T4	0.952 (0.728–1.244)	0.717			1.184 (0.912–1.536)	0.205		
Nodal stage	N0 + N1 versus N2a + N2b	1.162 (0.980–1.378)	0.084			1.245 (1.053–1.474)	**0.011**	1.119 (0.920–1.361)	0.260
Metastatic site	Brain + lung versus liver + others	1.540 (1.174–2.020)	**0.002**	1.536 (1.130–2.088)	**0.006**	1.728 (1.321–2.260)	<**0.001**	1.481 (1.094–2.006)	**0.011**
COX-2 expression	Negative versus positive	1.046 (0.779–1.406)	0.765			0.945 (0.704–1.270)	0.709		
Initial CEA (ng/mL)	<20 versus ≥20	3.103 (1.913–5.034)	<**0.001**	2.257 (1.366–3.730)	**0.001**	2.659 (1.641–4.310)	<**0.001**	1.838 (1.113–3.036)	**0.017**
MSI	MSI-H versus MSI-L/MSS	0.782 (0.493–1.238)	0.294			0.688 (0.435–1.089)	0.110		
C-MET expression	Negative/weak versus moderate/strong	1.690 (1.275–2.240)	<**0.001**	1.429 (1.052–1.940)	**0.022**	1.495 (1.129–1.979)	**0.005**	1.351 (0.993–1.839)	0.055
KRAS mutation	Yes versus no	2.112 (1.620–2.754)	<**0.001**	1.826 (1.361–2.450)	<**0.001**	2.050 (1.574–2.671)	<**0.001**	2.082 (1.545–2.805)	<**0.001**
BRAF mutation	Yes versus no	8.615 (5.537–9.045)	<**0.001**	4.798 (2.989–7.700)	<**0.001**	4.458 (2.935–6.771)	<**0.001**	3.864 (2.375–6.287)	<**0.001**
NRAS mutation	Yes versus no	0.620 (0.230–1.668)	0.344			0.462 (0.472–1.242)	0.126		
Anti-EGFR therapy	Yes versus no	0.599 (0.401–0.894)	**0.012**	0.742 (0.463–1.189)	0.215	0.694 (0.465–1.036)	0.074		
Bevacizumab therapy	Yes versus no	0.713 (0.529–0.963)	**0.027**	0.663 (0.469–0.937)	**0.020**	0.758 (0.562–1.022)	0.069	0.682 (0.484–0.961)	**0.029**
Surgery	Yes versus no	0.702 (0.532–0.927)	**0.013**	0.758 (0.531–1.082)	0.127	0.712 (0.539–0.941)	**0.017**	0.745 (0.525–1.057)	0.099

OS = overall survival; PFS = progression-free survival; HR = hazard ratio; CI = confidence interval; COX-2 = cyclooxygenase-2; C-MET = mesenchymal-epithelial transition factor; CEA = carcinoembryonic antigen; MSI = microsatellite instability; MSI-H = MSI-high; MSI-L = MSI-low; MSS = microsatellite stability.

**Table 5:** Treatment details of metastatic colorectal cancer patients.

Treatment methods	*n* (% of 461)	*n* (any mutation)	*n* (all wild-type)
Chemotherapy alone	159 (34.5%)	82	77
1 line	21 (4.6%)	15	6
2 lines	79 (17.1%)	52	27
≥3 lines	59 (12.8%)	15	44
Chemotherapy combined with surgery	118 (25.7%)	73	45
Primary lesion resection	63 (13.7%)	36	27
Metastasectomy	22 (4.8%)	16	6
Both	33 (7.2%)	21	12
Chemotherapy combined with radiotherapy	4 (0.8%)	2	2
Chemotherapy combined with targeted therapy	169 (36.6%)	65	104
Bevacizumab therapy	113 (24.5%)	61	52
Anti-EGFR therapy	52 (11.3%)	4	48
Both	4 (0.8%)	0	4
Chemotherapy combined with surgery and targeted therapy (primary lesion resection with anti-EGFR therapy)	2 (0.4%)	0	2
Chemotherapy for the entire population	452 (98.0%)	222	230
1 line	63 (13.7%)	48	15
2 lines	210 (45.5%)	119	91
≥3 lines	179 (38.8%)	55	124

## Supplementary Materials

Supplementary Materials Table 1: primer sequences and fragment length of RAS, BRAF, and EGFR gene amplification.
