# Supplementary material for: Predictive and Prognostic Implications of Mutation Profiling and Microsatellite Instability Status in Patients with Metastatic Colorectal Carcinoma
Source: Gastroenterol Res Pract. 2018 Jan 31;2018:4585802. doi: 10.1155/2018/4585802 (PMC5831938; doi:10.1155/2018/4585802)
Supplement: Supplementary Materials — Table 1: primer sequences and fragment length of RAS, BRAF, and EGFR gene amplification. [file 4585802.f1.docx]

Table 1: Primer sequences and fragment length of RAS, BRAF and EGFR genes amplification

| Exon | Forward Primer | Reverse Primer | Fragment length |
| --- | --- | --- | --- |
| KRAS |  |  |  |
| Exon 2 | CCAGACTGTGTTTCTCCCTTC | TTTAAACCCACCTATAATGGTG | 92bp |
| Exon 3 | CCAGACTGTGTTTCTCCCTT | CACAAAGAAAGCCCTCCCCA | 155bp |
| Exon 4 | TGATTTTGCAGAAAACAGAT | GACACAAAACAGGCTCAGGA | 120bp |
| BRAF |  |  |  |
| Exon 15 | TGCTTGCTCTGATAGGAAAA | AGCATCTCAGGGCCAAA | 119bp |
| NRAS |  |  |  |
| Exon 2 | ATGACTGAGTACAAACTGGTC | CTCTATGGTGGGATCATATTG | 128bp |
| Exon 3 | AAACAAGTGGTTATAGATGGT | CACAGAGGAAGCCTTCGCCT | 97bp |
| Exon 4 | ATGTGGCACCATCTCACATTA | GCACACAGCAAAGCAGAAAC | 122bp |
| HRAS |  |  |  |
| Exon 2 | CGGGGCCGAGGCCGGTGCGG | CCGGCCTCGGCCCCGGCCCT | 170bp |
| EGFR |  |  |  |
| Exon 19 | CATGTGGCACCATCTCACA | CCACACAGCAAAGCAGAAAC | 179bp |
| Exon 21 | CCTCACAGCAGGGTCTTCTC | TGCCTCCTTCTGCATGGTAT | 182bp |
